# Denoising Autoencoder, A Deep Learning Algorithm, Aids the Identification of A Novel Molecular Signature of Lung Adenocarcinoma

**DOI:** 10.1016/j.gpb.2019.02.003

**Published:** 2020-12-18

**Authors:** Jun Wang, Xueying Xie, Junchao Shi, Wenjun He, Qi Chen, Liang Chen, Wanjun Gu, Tong Zhou

**Affiliations:** 1 Department of Thoracic Surgery, Jiangsu Province People’s Hospital and the First Affiliated Hospital of Nanjing Medical University, Nanjing 210029, China; 2 State Key Laboratory of Bioelectronics, School of Biological Sciences and Medical Engineering, Southeast University, Nanjing 210096, China; 3 Department of Physiology and Cell Biology, University of Nevada, Reno School of Medicine, Reno, NV 89557, USA; 4 State Key Lab of Respiratory Disease, Guangzhou Medical University, Guangzhou 510000, China

**Keywords:** Denoising autoencoder, Unsupervised learning, Lung cancer, Prognosis, Molecular signature

## Abstract

Precise biomarker development is a key step in disease management. However, most of the published biomarkers were derived from a relatively small number of samples with supervised approaches. Recent advances in unsupervised machine learning promise to leverage very large datasets for making better predictions of disease biomarkers. **Denoising autoencoder** (DA) is one of the unsupervised deep learning algorithms, which is a stochastic version of autoencoder techniques. The principle of DA is to force the hidden layer of autoencoder to capture more robust features by reconstructing a clean input from a corrupted one. Here, a DA model was applied to analyze integrated transcriptomic data from 13 published **lung cancer** studies, which consisted of 1916 human lung tissue samples. Using DA, we discovered a **molecular signature** composed of multiple genes for lung adenocarcinoma (ADC). In independent validation cohorts, the proposed molecular signature is proved to be an effective classifier for lung cancer histological subtypes. Also, this signature successfully predicts clinical outcome in lung ADC, which is independent of traditional prognostic factors. More importantly, this signature exhibits a superior prognostic power compared with the other published prognostic genes. Our study suggests that **unsupervised learning** is helpful for biomarker development in the era of precision medicine.

## Introduction

Lung cancer is the most frequently diagnosed cancer and the leading cause of cancer death all over the world [1], [2]. Based on the size and appearance of the malignant cells, lung cancers are mainly classified into non-small-cell and small-cell lung cancers [3]. Lung adenocarcinoma (ADC), the most common subtype of non-small-cell lung cancers originating from peripheral lung tissue, accounts for nearly 40% of all lung cancers [3]. To gain better lung ADC diagnosis, prognosis, and treatment, high-throughput molecular profiling methods have been used to characterize lung ADC in recent years [4]. Using whole-genome sequencing and/or whole-exome sequencing methods, several studies have reported several somatic mutations, structural rearrangements, and copy number variations related to key biological pathways in lung ADC [5], [6], [7], [8], [9], [10], [11], [12], [13]. In addition, Liu et al. [10] identified 106 splice-site mutations associated with cancer-specific aberrant splicing using both whole-genome sequencing and transcriptome sequencing methods. White et al. [14] also identified several differentially expressed long intergenic non-coding RNAs in lung ADC. Combinations of mRNA, microRNA, and DNA sequencing with copy number, methylation, and proteome analyses revealed a comprehensive molecular profiling of lung ADC [12]. Based on these molecular profiling data and the clinical phenotype data, many biomarker sets have been identified that provide better diagnosis or prognosis of lung ADC [15], [16], [17], [18], [19], [20], [21], [22], [23]. Specifically, Okayama et al. [20] developed a prognostic classifier, which consists of the expression levels of four genes to identify stage I lung ADC, and has been validated in five independent cohorts [20]. Our previous work also identified the expression levels of 37 ion channel genes to predict survival in lung ADC [17]. In the same study, we proposed another set of 13 ion channel genes an overall diagnostic biomarker set to differentiate lung cancer subtypes [17]. These studies provide a foundation for classification, outcome prediction, and treatment guidance of lung ADC.

Although substantial improvements have been made in the past several years, the diagnosis, prognosis, and treatment of lung ADC are far from precise [4]. In the era of precision medicine, efficient biomarker identification is a fundamental necessity; a lack of such biomarkers is an obstacle to improving the precision of disease management [23]. Traditional lung ADC biomarkers were normally derived from a relatively small cohort size, which may cause the population bias observed with previously identified biomarkers [23]. To overcome the drawback of these traditional biomarkers, an ideal precision medicine research is to increase the magnitude of data collected and to analyze them simultaneously [23]. Fortunately, a large amount of molecular profiling data, such as gene expression data of lung ADC, are available in public databases. Some studies have identified novel regulators and potential targets of lung ADC by integrating these data from various sources. For example, Chen et al. [24] analyzed 13 gene expression datasets using a meta-analysis approach and identified *PTK7* as a survival gene in lung ADC. More thorough analysis, however, is needed to identify novel and useful biomarkers from the huge amount of data to manage lung ADC.

Notably, recent advances in machine learning methods, such as deep learning, have promised to leverage very large datasets for making better inferences [25], [26]. Using deep neural networks, several studies have exhibited good accuracy in predicting splicing patterns [27], sequence specificities of DNA- and RNA-binding proteins [28], and functional effects of non-coding variants [29], [30]. These studies hint that the deep learning method possesses promising power in integrating large biological datasets to make inferences, and that deep learning could be a useful algorithm to identify biomarkers from large-scale gene expression datasets. Some pioneer studies have also successfully applied deep learning algorithms in analyzing whole transcriptome data. For example, a multi-task, multi-layer, feed-forward neural network was developed to infer the expression of target genes from the expression of some landmark genes [31]. Some autoencoder models were used to extract meaningful features from whole genome-scale gene expression data [32], [33], [34]. The denoising autoencoder (DA) model is a stochastic version of the autoencoder techniques. The principle of DA is simple: in order to force the hidden layer of autoencoder to capture more robust features, we train the autoencoder to reconstruct a clean (repaired) input from a partially destroyed (corrupted) input, which is motivated by the rationale that “a good representation is one that can be obtained robustly from a corrupted input and that will be useful for recovering the corresponding clean input” [35]. In this study, we hypothesize that the DA model is useful in constructing meaningful features related to disease classification and survival prediction from large-scale transcriptome data in lung ADC. We integrated the genome-wide expression data from 13 published lung cancer studies, which consisted of 1916 human lung tissue samples, and applied a DA model to analyze this large dataset. We next identified some important DA hidden nodes that were related to clinical phenotypes and constructed a molecular signature composed of multiple genes from the hidden DA nodes. Using independent validation cohorts, we confirmed that the proposed molecular signature potentially serves as a classifier for lung cancer histological subtypes. Also, this signature successfully predicts clinical outcome in lung ADC, which is independent of traditional prognostic factors.

## Results

### Constructing the DA model

**Figure 1:**
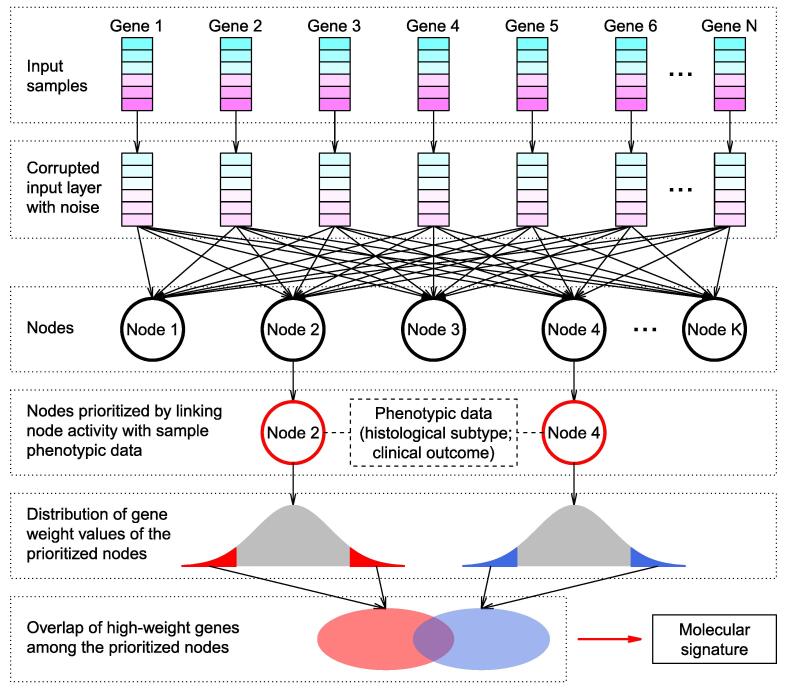
**Schematic of the strategy to identify the molecular signature** The expression information of N genes was the input of the denoising autoencoder (DA) model. Corrupted input was generated by randomly adding noise to the original gene expression data. A hidden layer with K nodes was then constructed by autoencoder. Each node was connected to each gene. The values contained in each node were termed node activity. By linking node activity with sample phenotypic data (*e.g.*, tumor histological subtype and clinical outcome), significant nodes were prioritized, that is, Node 2 and Node 4 in this schematic. Within each node, each gene was assigned a weight reflecting the contribution of the gene to the node activity. Genes with weight within both tails of the weight distribution were defined as high-weight genes. The overlapping high-weight genes among the prioritized nodes were finally defined as signature genes.

We obtained 13 lung cancer transcriptome datasets from the Gene Expression Omnibus (GEO) database [36], which were all based on the Affymetrix Human Genome U133 Plus 2.0 Array (Table S1). In total, 1916 human lung tissue samples were collected, including 224 control (CTR) samples, 827 samples from ADC patients, 357 samples from patients with squamous-cell carcinoma (SCC), 76 samples from patients with large-cell carcinoma (LCC), 21 samples from patients with small-cell lung carcinoma, 2 samples from adenosquamous carcinoma patients, 39 samples from basaloid carcinoma patients, 24 samples from patients with carcinoid tumor, 56 samples from patients with large cell neuroendocrine carcinoma, and 290 samples without clear classification information. We used the ADAGE package [33] to construct the DA model as illustrated in Figure 1 (see Materials and methods for details). The microarray probeset expression data of all these samples were used as training input of DA. By adding random noise to the input expression values, corrupted expression data were constructed, which were next encoded into 200 nodes. All probesets were connected to each node by a weight vector, which measures the contribution of each probeset to the node. The node activity of each sample was further computed as the inner product between the corrupted input of the sample and the weight vector. The probesets with extreme positive or negative weights were considered as high-weight probesets, which provide the strongest impact to the node activity.

### The histological subtype-associated nodes

**Figure 2:**
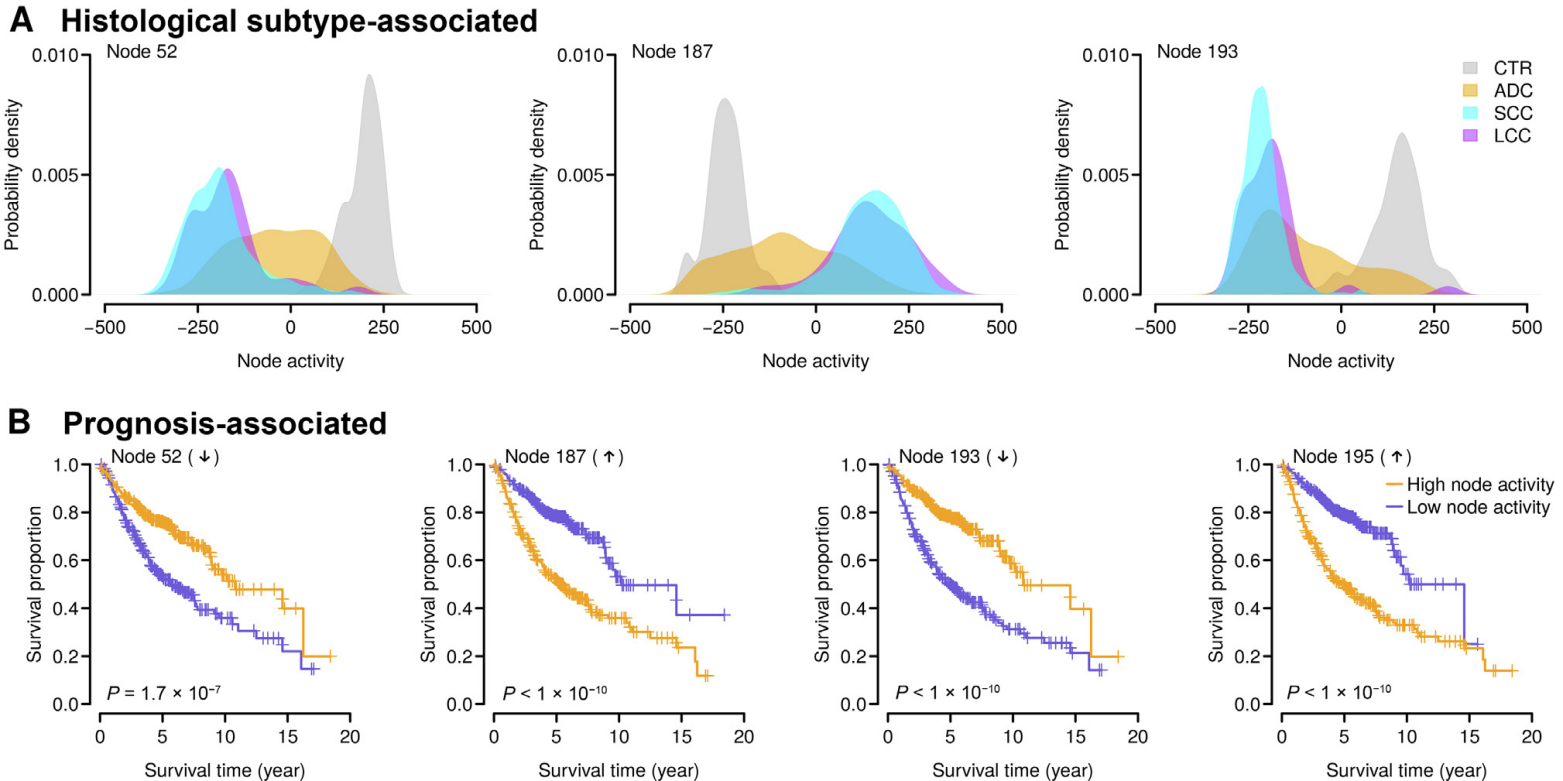
**Identifying clinically relevant nodes** **A.** The top nodes distinguishing the adenocarcinoma (ADC) patients from the control (CTR), squamous-cell carcinoma (SCC), and large-cell carcinoma (LCC) subjects. Each panel indicates the distribution of node activity in each category. **B.** The top nodes significantly associated with clinical outcome in the ADC patients. The overall survival data were analyzed here. The activities of Node 52 and Node 193 (negative nodes) were decreased in the ADC patients with poorer overall survival, while the activities of Node 187 and Node 195 (positive nodes) were increased in the ADC patients with poorer overall survival. The median node activity was used as a cutoff to separate the high node activity and low node activity groups. The *P* values were computed by log-rank test for the difference in survival between the two groups.

We next assessed the association of each node with patient phenotypic information. To avoid the bias caused by batch effect, one-way ANOVA was performed to test the difference in node activity among different datasets. The nodes with the top 100 largest *F*-statistic were excluded. We also removed the nodes with activity variance smaller than 0.002 (node activity followed a bimodal distribution and 0.002 was the pit between the peaks in the probability density function). The retained nodes were subject to comparison regarding histological subtypes. Due to sample size limitation for some rare histological subtypes, we only focused on the ADC, SCC, LCC, and CTR samples in this study. The node activity of the ADC samples was compared to that of the CTR, SCC, and LCC samples, respectively. For each type of comparison (*i.e.*, ADC *vs.* CTR, ADC *vs.* SCC, or ADC *vs.* LCC), all the nodes were ranked according to the *P* values computed by *t*-test and only the nodes with the top five lowest *P* values were retained. In total, we identified three nodes, Node 52, Node 187, and Node 193, among the top five in all three comparisons (Figure 2A). The activities of these three nodes significantly differentiated the ADC samples from the CTR, SCC, and LCC samples (*t*-test, *P* < 1 ×  10^−10^ in all three comparisons), which potentially serves as a predictor of histological subtypes (Figure 2A, Figure S1).

### The prognosis-associated nodes

We also investigated the association between node activity and clinical outcome of the ADC patients. Among the 827 ADC samples in the training set, overall and recurrence-free survival data were available for 615 and 519 subjects, respectively. Univariate *Cox* proportional hazards regression was applied to evaluate the relationship between ADC outcome and activity of each node. All the nodes were then ranked based on the *P* values computed by *Cox* regression. Consequently, we identified four nodes, Node 52, Node 187, Node 193, and Node 195, in which node activity was strongly associated (within the top five) with both overall and recurrence-free survival. The activities of Node 52 and Node 193 were significantly downregulated [*Cox* regression: *P* = 1.7 × 10^−7^ (overall survival) and *P* < 1 × 10^−10^ (recurrence-free survival) for Node 52; *P* < 1 ×  10^−10^ (overall survival) and *P* = 2.9 × 10^−^^6^ (recurrence-free survival) for Node 193] in the ADC patients with poorer survival (Figure 2B, Figure S2). By contrast, the activities of Node 187 and Node 195 were significantly upregulated [*Cox* regression: *P* < 1 ×  10^−10^ (overall survival) and 3.8 × 10^−^^5^ (recurrence-free survival) for Node 187; *P* < 1 ×  10^−10^ (overall survival) and 7.9 × 10^−^^6^ (recurrence-free survival) for Node 195] in the ADC patients with poorer survival (Figure 2B, Figure S2).

### The construction of a 35-gene signature

**Figure 3:**
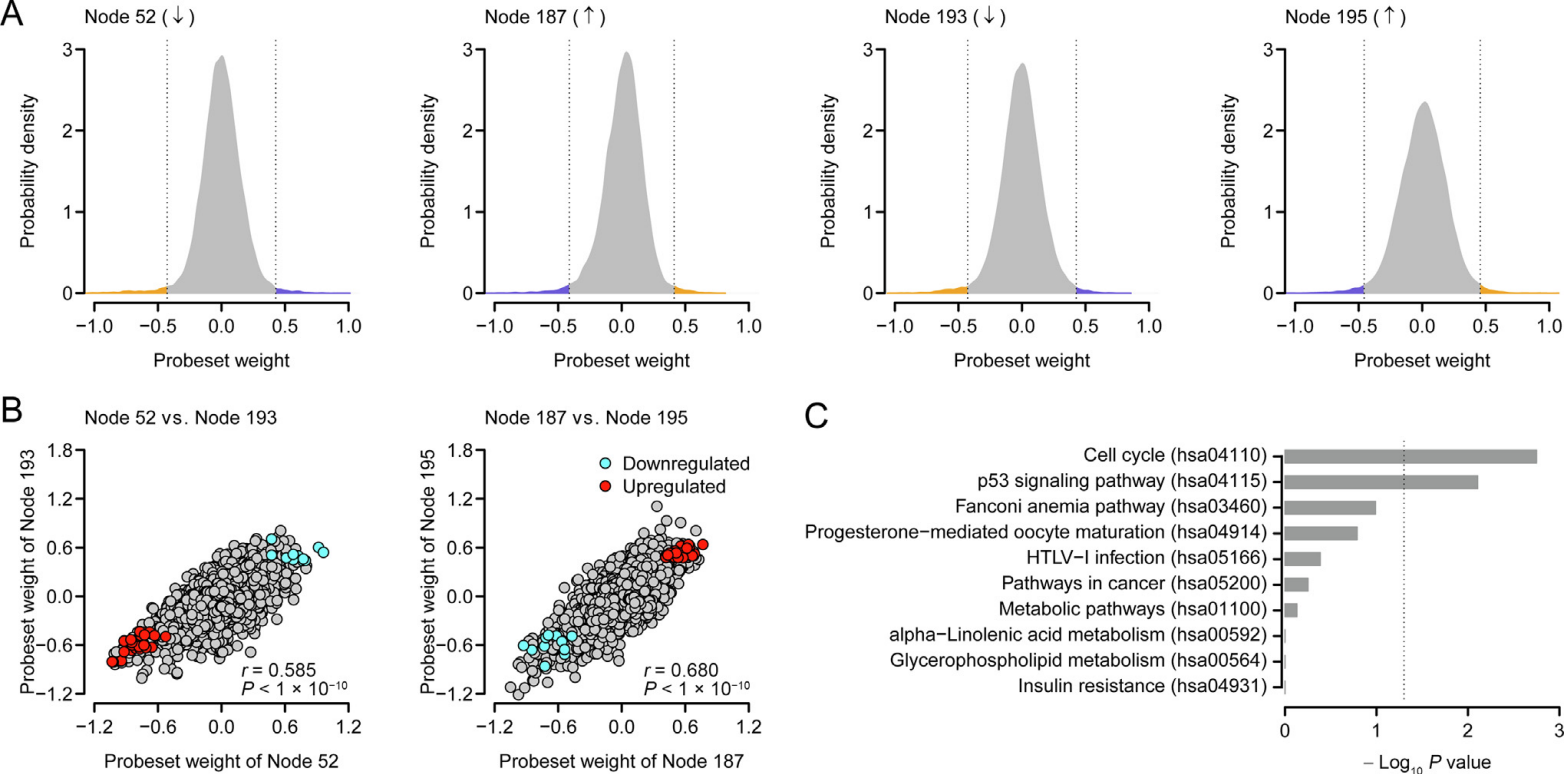
**Prioritizing high-weight probesets** **A.** Distribution of probeset weight in the prioritized nodes. The orange areas represent the high-weight probesets (within 1% tail) upregulated in the patients with poorer survival, whereas the blue areas denote the high-weight probesets (within 1% tail) downregulated in the patients with poorer survival. **B.** Correlation in probeset weight between the prioritized nodes. The red and blue dots represent the overlapping upregulated and downregulated probesets in the patients with poorer survival, respectively. **C.** The top Kyoto Encyclopedia of Genes and Genomes (KEGG) pathways associated with the signature genes. The *P* values were calculated by Fisher’s exact test. The vertical dash line denotes the significance level of *α* = 0.05.

DA nodes are derived from the expression values of the human transcriptome (Figure 1), which can be used directly for diagnostic or prognostic purpose as suggested by Tan et al. [32], [33]. In contrast, to build mRNA-based biomarkers, we selected a small number of human genes that can predict the survival of human lung ADC (Figure 1). Here, we designated Node 52 and Node 193 as “negative nodes” and Node 187 and Node 195 as “positive nodes”. The probeset weights in the four prioritized nodes followed a bell-shape distribution (Figure 3A). Because the node activity of each specific sample was the inner product of the probeset weight vector of the node and the corrupted probeset expression of the sample, the high-weight probesets (within either left or right 1% tail; Figure 3A) in each prioritized node exhibited the most influence over the node activity. The high-negative-weight probesets in the negative nodes and the high-positive-weight probesets in the positive nodes (the orange areas in Figure 3A) were potentially upregulated in the patients with poorer survival, while the high-positive-weight probesets in the negative nodes and the high-negative-weight probesets in the positive nodes (the blue areas in Figure 3A) were downregulated in the patients with poorer survival. As expected, strong positive correlation in probeset weight was observed between the two negative nodes (Node 52 *vs.* Node 193) and between the two positive nodes (Node 187 *vs.* Node 195) (Figure 3B). On the contrary, the probeset weights between positive and negative nodes were negatively correlated (Figure S3). We next focused on the intersection of the high-weight probesets among the four prioritized nodes. In total, we identified 40 overlapping probesets within the intersection, including 29 upregulated and 11 downregulated probesets in the patients with poorer survival (Figure 3B), which were mapped to 35 unique well-annotated human genes. We designated these 35 genes as the 35-gene signature (Table S2). A weight was assigned to each gene within the signature: 1 and –1 for the genes positively and negatively associated with worse prognosis, respectively. Kyoto Encyclopedia of Genes and Genomes (KEGG) pathway enrichment analysis demonstrated that the 35-gene signature was significantly associated with some cancer-related KEGG terms, *e.g.*, “p53 signaling pathway” (Figure 3C).

Actually, among the four prognosis-related nodes, three nodes (*i.e.*, Node 52, Node 187, and Node 195) were also the nodes that best classified histological subtypes (Figure 2A). Therefore, it is reasonable to hypothesize that the 35-gene signature can be used for both diagnostic (distinguishing ADC patients from non-ADC subjects) and prognostic (predicting clinical outcome for ADC patients) purposes.

### The 35-gene signature distinguishes ADC patients from non-ADC samples

**Figure 4:**
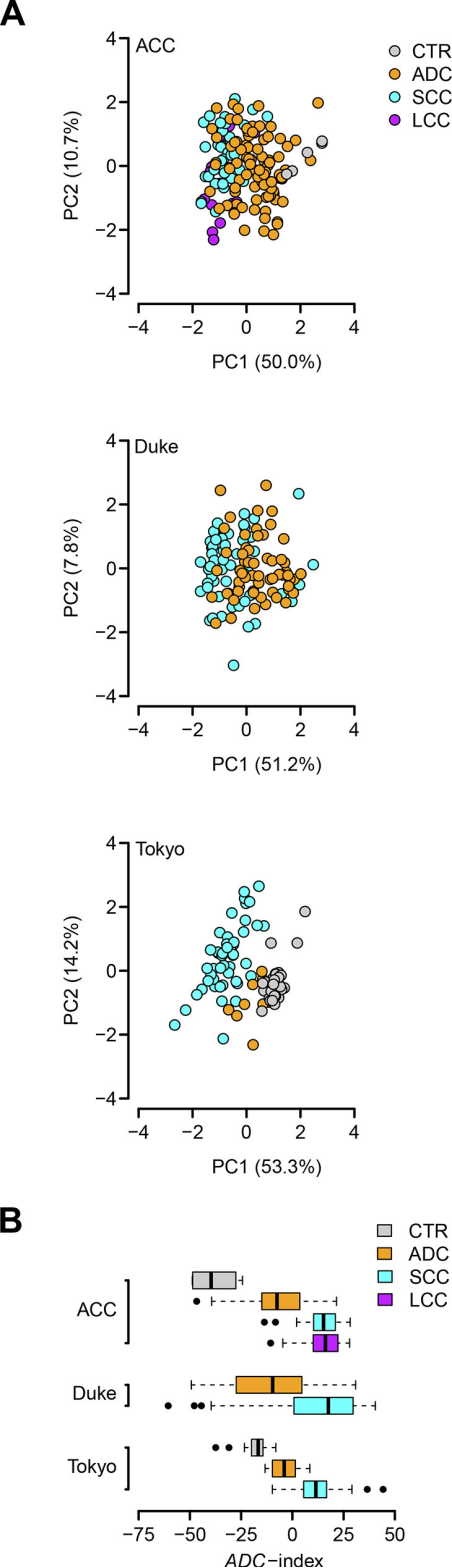
**The 35-gene signature distinguishes ADC patients from non-ADC subjects in the validation cohorts** **A.** Principal component analysis (PCA) on the 35-gene signature in the Aichi Cancer Center (ACC), Duke University Medical Center (Duke), and University of Tokyo (Tokyo) cohorts. PC1, the first principal component; PC2, the second principal component. ACC, the cohort from Aichi Cancer Center, Japan (GEO: GSE11969) [37]; Duke, the cohort from Duke University Medical Center, USA (GEO: GSE3141) [38]; Tokyo, the cohort from University of Tokyo, Japan (GEO: GSE2088) [39]. **B.** The 35-gene based *ADC*-index differentiates the ADC patients from the CTR, SCC, and LCC subjects in the validation cohorts.

To validate the diagnostic role of the 35-gene signature, we investigated its classification performance in three independent validation cohorts from Aichi Cancer Center (ACC), Japan (GEO: GSE11969) [37], Duke University Medical Center (Duke), USA (GEO: GSE3141) [38], and University of Tokyo (Tokyo), Japan (GEO: GSE2088) [39], respectively. There are 5 CTR samples, 90 ADC patients, 35 SCC patients, and 18 LCC patients in the ACC cohort; the Duke cohort is composed of 58 ADC patients and 53 SCC patients; the Tokyo cohort includes 30 CTR samples, 9 ADC patients, and 48 SCC patients. Principal component analysis (PCA) indicates that the 35-gene signature differentiates ADC patients from non-ADC samples in all the validation cohorts (Figure 4A). To statistically assess the classification power of the 35-gene signature, a classification index (*ADC*-index) was assigned to each human subject (see Materials and methods for details). *ADC*-index is a linear combination of the gene expression values of the 35 genes in the 35-gene signature. Firstly, the *ADC*-index was significantly higher in the ADC patients than in the CTR samples in the ACC and Tokyo cohorts (*t*-test, *P* = 0.003 for the ACC cohort and *P* = 3.6 × 10^−4^ for the Tokyo cohort; Figure 4B). Secondly, the *ADC*-index of the ADC patients was significantly lower than that of the SCC patients in all the validation cohorts (*t*-test, *P* < 1 ×  10^−10^ for the ACC cohort, *P* = 8.5 × 10^−7^ for the Duke cohort, and *P* = 6.6 × 10^−5^ for the Tokyo cohort; Figure 4B). Thirdly, the *ADC*-index was also significantly decreased in the ADC patients compared with the LCC patients in the ACC cohort (*t*-test, *P* = 1.3 × 10^−8^; Figure 4B). All these results strongly suggest that the 35-gene based *ADC*-index potentially serves as a predictor of histological subtypes.

### The 35-gene signature predicts clinical outcome for ADC patients

**Figure 5:**
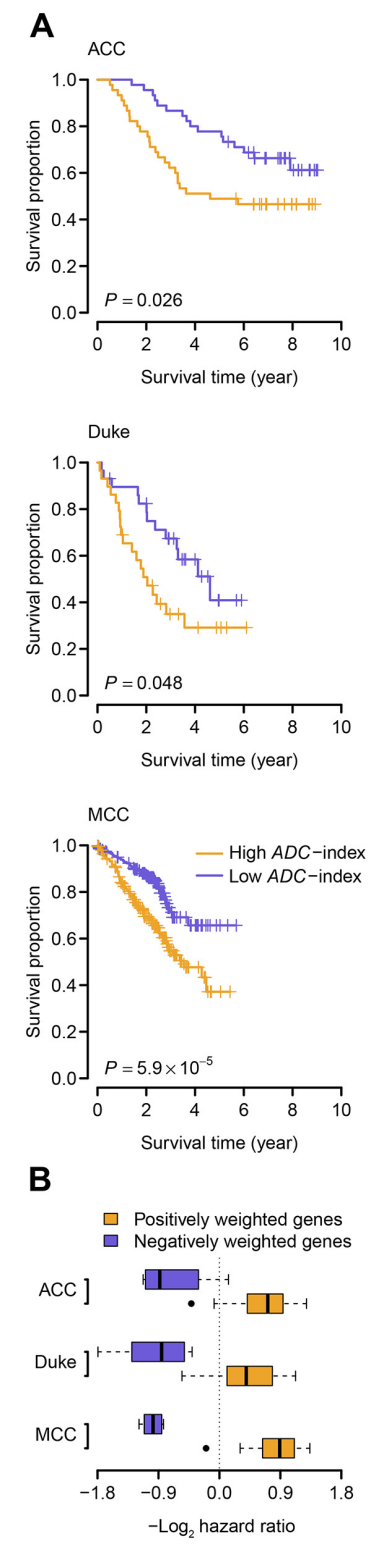
**The 35-gene based *ADC*-index predicts overall survival in the validation cohorts** **A.***Kaplan–Meier* curves for the ADC patients in the three validation cohorts. Patients were stratified into two categories according to *ADC*-index. The median *ADC*-index was used as a cutoff. *P* values indicate significant differences in overall survival as measured by log-rank test. **B.** Boxplot of hazard ratio of the genes within the 35-gene signature. For each gene, the ADC patients were stratified into two groups using the median expression value of the gene as a cutoff. Hazard ratio was computed between the two groups (high-expression over low-expression). The hazard ratios of the positively weighted genes are significantly higher than those of the negatively weighted genes. MCC, the cohort from Moffitt Cancer Center, USA (GEO: GSE72094) [40].

We next tested whether the 35-gene based *ADC*-index can predict survival for ADC patients. Besides the ACC and Duke cohorts, we analyzed one more validation cohort from Moffitt Cancer Center (MCC), USA (GEO: GSE72094) [40], which consists of 442 ADC patients (survival data are available for 398 patients). Univariate *Cox* proportional hazards regression of survival indicates that *ADC*-index is positively associated with worse survival for the ADC patients from the validation cohorts, except for the Duke cohort (Table 1). Increase by one in *ADC*-index enhances the risk of death by 4%, 1%, and 1% for the ACC, Duke, and MCC cohorts, respectively (Table 1). Using the median *ADC*-index as a cutoff, we further stratified the ADC patients into two groups for each validation cohort. *Kaplan–Meier* survival curves demonstrated a significant difference in survival between the two patient groups in all three validation cohorts (log-rank test, *P* = 0.026 for the ACC cohort, *P* = 0.048 for the Duke cohort, and *P* = 5.9 × 10^−5^ for the MCC cohort; Figure 5A).

**Table 1:** **Univariate *Cox* proportional hazards regression of survival by *ADC*-index**

*Note*: HR, hazard ratio; CI, confidence interval; ADC, adenocarcinoma; SCC, squamous-cell carcinoma; LCC, large-cell carcinoma; ACC, the cohort from Aichi Cancer Center, Japan (GEO: GSE11969) [37]; Duke, the cohort from Duke University Medical Center, USA (GEO: GSE3141) [38]; MCC, the cohort from Moffitt Cancer enter, USA (GEO: GSE72094) [40].

We also investigated the prognostic patterns of the individual genes within the 35-gene signature. For each gene, the ADC patients were stratified into two groups using the median expression value of the gene as a cutoff. The hazard ratio of death was then computed between the two patient groups (high-expression group over low-expression group). We found that, in all the validation cohorts, the hazard ratios of the positively weighted genes were significantly higher than those of the negatively weighted genes (*t*-test, *P* = 2.2 × 10^−5^ for the ACC cohort, *P* = 6.1 × 10^−6^ for the Duke cohort, and *P* < 1 ×  10^−10^ for the MCC cohort; Figure 5B). The expression of the genes with a positive weight tends to be positively correlated with worse prognosis (*i.e.*, hazard ratio > 1), whereas the negatively weighted genes tend to have a hazard ratio < 1 (Figure 5B). These results confirm the robustness of the 35-gene signature.

Finally, we tested the prognostic power of the 35-gene based *ADC*-index in SCC and LCC patients, respectively. We failed to identify, however, any significant association between *ADC*-index and clinical outcome in SCC and LCC (Table 1; Figure S4). This suggests that the 35-gene signature is an ADC-specific prognostic predictor.

### The 35-gene based *ADC*-index is independent of standard prognostic covariates

To confirm the role of the 35-gene signature as an independent prognostic factor, multivariate *Cox* model was applied to investigate the performance of *ADC*-index in comparison with the traditional prognostic variables in lung cancer, including age, gender, smoking history, grade, stage, and mutation statuses of *EGFR*, *KRAS*, *STK11*, and *TP53*. Because of the limited phenotypic information in the Duke cohort, only the ADC patients from the ACC and MCC cohorts were considered here. Multivariate *Cox* proportional hazards regression of survival indicates that the 35-gene based *ADC*-index remains a significant covariate in relation to the traditional clinical factors in both ACC and MCC cohorts (*P* = 0.003 for the ACC cohort and *P* = 6.5 × 10^−5^ for the MCC cohort; Table 2), which suggests that the 35-gene based *ADC*-index is an independent prognostic variable.

**Table 2:** **Multivariate *Cox* proportional hazards regression of survivalin ADC patients**

### Superior prognostic power of the 35-gene signature

**Figure 6:**
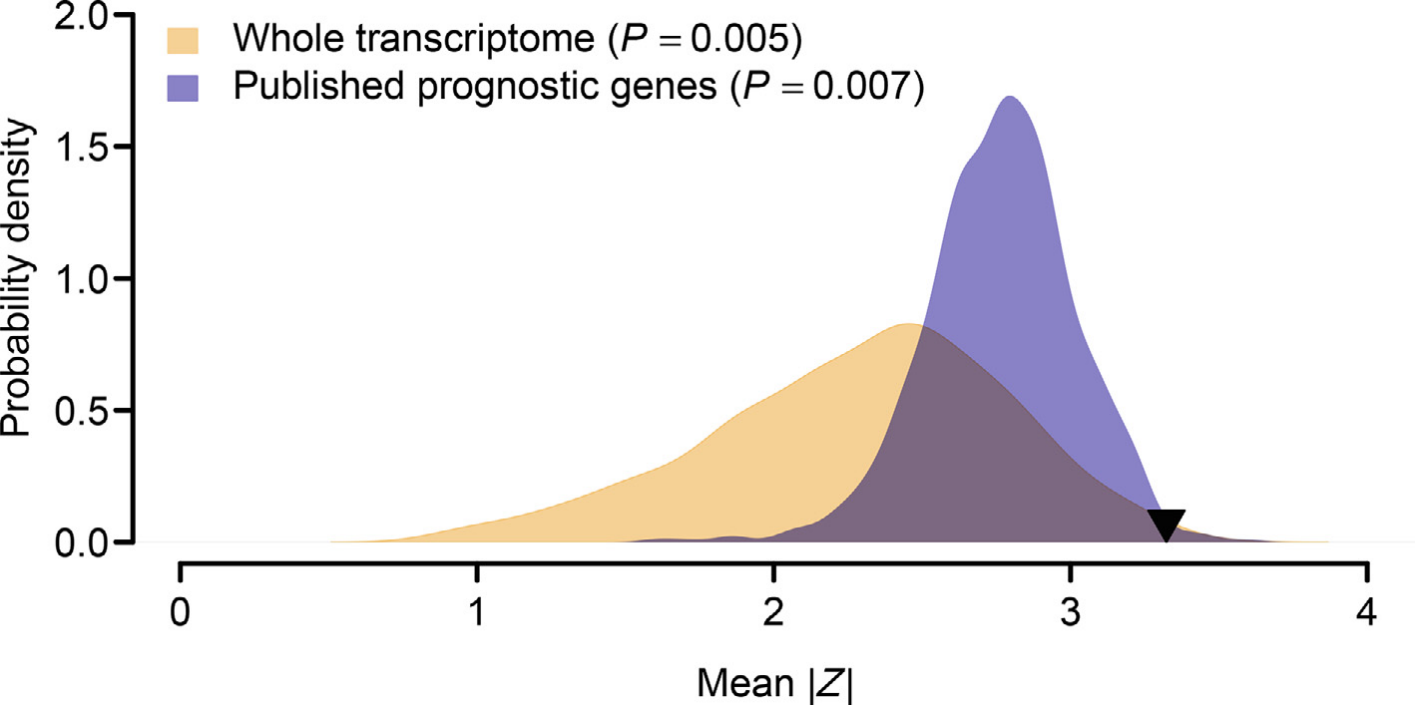
**Superior prognostic power of the 35-gene signature** The orange area shows the distribution of the mean of |*Z*| for the 1000 resampled gene signatures (with 35 genes for each gene signature) randomly picked up from human whole transcriptome. The blue area shows the distribution of the mean of |*Z*| for the 1000 resampled gene signatures (with 35 genes for each gene signature) randomly selected from the pool of the published prognostic genes. The black triangle stands for the mean of |*Z*| of the 35-gene signature. Right-tailed *P* value was computed for each resampling test.

It was reported that the prognostic power of some published gene signatures is not significantly better than that of random gene sets with identical size [41]. Therefore, we followed the resampling procedures suggested by Venet et al. [41] to test whether the 35-gene signature performed better than random signatures. We artificially constructed 1000 random gene signatures with identical size as the 35-gene signature. Both PCA and *Cox* regression were conducted for each artificially resampled signature. The association between the first principal component and clinical outcome was recorded as the average absolute value of *Cox Wald* statistic (|*Z*|) in the three validation cohorts. We found that the mean of |*Z*| of our real signature was significantly larger than that of the artificial gene signatures (right-tailed, *P* = 0.005; Figure 6), which suggests a non-random prognostic power of the 35-gene signature.

We next compared the prognostic power of the 35-gene signature against the published lung cancer prognostic genes. In total, 425 prognostic genes were collected from previous studies [42], [43], [44], [45], [46], [47], [48], [49], [50], [51], [52]. We performed a resampling test to check whether the prognostic power of the 35-gene signature was statistically better than the other prognostic genes. For each round of randomization, 35 genes were randomly picked up from the pool of the published prognostic genes. We found that the mean of |*Z*| of the 35-gene signature was significantly larger than that of the 1000 random gene signatures consisting of published prognostic genes (right-tailed, *P* = 0.007; Figure 6), which suggests the superior prognostic power of the 35-gene signature.

To test the robustness of DA, we further built 100 DA models at size of 200 and 400 nodes, respectively, using different seeds, which resulted in DA models with different local minima. We applied the same procedures as above described to prioritize gene signatures from different DA models. The genes identified by individual DA models were pooled together. In total, 272 and 371 unique genes were collected from the 100 DA models of 200 nodes and the 100 DA models of 400 nodes, respectively, which significantly overlapped with the 35-gene signature (33 and 35 overlapping genes for 200-node and 400-node models, respectively; hypergeometric test, *P* < 1 ×  10^−10^). We next performed a resampling test with 1000 rounds to check whether the prognostic power of the DA genes was statistically better than the published lung cancer prognostic genes. For each round of randomization, 35 genes were randomly picked up from the pool of the DA genes prioritized by the 200-node and 400-node models, respectively. We found that the mean of |*Z*| of both the 200-node and 400-node DA genes was significantly larger than that of the 1000 random gene signatures consisting of published prognostic genes (*t*-test, *P* < 1 ×  10^−10^; Figure S5). Interestingly, the prognostic power of the genes collected from the 200-node DA models was significantly better than that of the genes collected from the 400-node models (*t*-test, *P* < 1 ×  10^−10^; Figure S5). To compare with linear approaches, we also extracted 261 prognostic genes using PCA-based method (see Materials and methods for details). Resampling test demonstrated that the mean of |*Z*| of the PCA genes was significantly lower than that of both the 200-node and 400-node DA genes (*t*-test, *P* < 1 ×  10^−10^; Figure S5). All these results suggest that the DA-based framework can robustly generate molecular signatures from transcriptomic data and larger node number does not necessarily improve DA reconstruction [53].

## Discussion

In this study, we successfully applied an unsupervised DA-based framework, a deep learning algorithm, to extract important features from large scale of high-dimensional genome-wide expression data. Unlike some unsupervised algorithms used in feature extraction, such as PCA, independent component analysis, and cluster algorithms, which consider linear mappable information from input to features, DAs can capture higher-level structures from the observed input in the non-linear space [32], [33]. In other words, DA can be deemed as a non-linear generalization of linear models and can grasp the higher-level and complex correlations from low-level features. For example, Hinton et al. [54] indicates that autoencoder, which is the core algorithm of DA, performs much better than PCA as a tool to learn low-dimensional codes. By introducing random noises into real expression inputs in model training process, DA can robustly extract stable biological principles among genes from genome-wide expression data [32], [33]. We trained a DA model with 200 output nodes from 1916 microarray gene expression datasets (Figure 1). From these output nodes, we identified three top nodes that can discriminate ADC samples from CTR, SCC, and LCC samples without referring to any prior knowledge on the phenotypic data (Figure 2A). Similarly, we also detected four nodes that are significantly related to both overall and recurrence-free survival in ADC patients (Figure 2B). These results suggest that DA model can successfully extract some low-dimensional molecular features that are related to both disease diagnosis and prognosis from large-scale genome-wide expression datasets. Notably, these identified nodes are independent from how the datasets are generated. This is especially important when the datasets are integrated from various sources, as batch effects inherently exist in the dataset, which will skew the derived conclusions [55]. The superior performance of the DA model on human lung ADC confirms its potential application in biomarker development, especially when the datasets are larger and integrated from various sources.

Interestingly, all the three nodes that can separate ADC from CTR, SCC, and LCC samples (Node 52, Node 187, and Node193) are among the top five predictors of lung ADC survival (Figure 2A and B). The overlap between the ADC diagnostic and prognostic nodes is remarkable, which suggests that a single set of marker genes may serve a dual purpose. Also, the overlap suggests that these three nodes learned a combined transcriptome pattern that captures both histological and prognostic features of ADC, which may be missed by existing methods used in biomarker development [23]. Further efforts are required to investigate the underlying causes and to explore if it is general for other cancers.

By overlapping upregulated and downregulated genes with high-weight connection to four nodes, we developed a 35-gene signature as a diagnostic and prognostic biomarker for human lung ADC (Table S2). These 35 genes are significantly involved in some cancer-related pathways, such as “p53 signaling pathway” (Figure 3C). Among them, some genes have been identified to be related to human lung ADC in previous studies. For example, the expression value of *SCN7A* was identified to be negatively associated with ADC survival in this study (Table S2), which is consistent with our previous results that *SCN7A* expression was increased in lung ADC [56]. Some other genes, such as *BIRC5* [57], *BLM* [58], and *CCNB2* [59], are also differentially expressed in non-small-cell lung cancer. However, most genes in the 35-gene signature are first reported to classify the human lung cancer subtypes and to predict the survival outcome of lung ADC patients. This finding is not surprising, be the majority of previous studies either relied on the prior knowledge to choose some relevant genes for goal-directed experiments or carried out some small-scale data analyses limited to the methods [16], [17], [60], [61]. Unlike the traditional methods used before, the DA framework used in our study can integrate a large number of available datasets. The framework learns their intrinsic stable structures without any predefined knowledge and is only dependent on the source data. The algorithm of the DA model and the integrated larger datasets assure that the identified 35-gene signature is a novel diagnostic and prognostic biomarker for human lung ADC.

We also evaluated the molecular classification and survival outcome prediction performance of the 35-gene signature in several independent cohorts. Our results show that the 35-gene based *ADC*-index can significantly separate human lung ADC patients from non-ADC samples in ACC, Duke, and Tokyo cohorts (Figure 4). Furthermore, *ADC*-index is also significantly associated with worse survival of ADC patients in ACC and MCC cohorts (Table 1). These results suggest that the 35-gene signature is a universal diagnostic and prognostic biomarker for different population cohorts. This observation is reasonable, because the 35-gene signature that we proposed is learned from a larger dataset that integrates human lung ADC samples from 13 published studies (Table S1). Given that the samples of those 13 studies are collected from different areas of the world, the 35-gene signature should be independent from the population cohorts. Further analysis indicates that *ADC*-index and cancer stage are the two independent indicators of survival outcome for human lung ADC patients in both ACC and MCC cohorts (Table 2). Some other potential factors, however, such as age, smoking history, and mutational statuses of some cancer genes, are not consistently related to disease outcome in human lung ADC (Table 2). Cancer stage is closely related to cancer survival in human lung ADC [62], but our results suggest that gene expression values of those 35 signature genes can contribute another dimension of knowledge on ADC survival outcome. The combination of cancer stage and *ADC*-index should offer better prognostic information of cancer outcome for human lung ADC.

## Conclusion

The DA model, a deep learning algorithm, can be used to dissect important features from genome wide-expression datasets of human lung cancers. Some of the features are closely related to sample phenotypic information, such as cancer subtypes and disease outcome. By focusing on those phenotype-related features, a 35-gene signature has been constructed. This molecular signature is further validated to be a good diagnostic and prognostic biomarker of human lung ADC in several independent validation cohorts. This method we show here is proved to be an effective way to analyze large integrated datasets from various studies, which should be useful in developing precise biomarkers in the precision medicine era.

## Materials and methods

### The DA model

The DA model was constructed using the ADAGE package developed by Tan et al. [33], which summarizes the genome-wide gene expression profiles in human lung tissues into clinically relevant features. Firstly, random noise was added to the input expression data (Figure 1). Secondly, the neural networks with hidden nodes were trained by the corrupted input to remove the added noise and recover the original undistorted input, which potentially discovers more robust features. All genes were connected to each hidden node through a weight vector, which measures the contribution of all the individual genes to the node. The constructed feature of each node can be reflected by the node activity of each sample, which is the sigmoid transformation of a bias vector plus the inner product between the corrupted input of the sample and the weight vector. The sigmoid function is widely used in DA implementation (and many other machine learning algorithms as well), which can capture the complex non-linear relationship in the high-dimensional data. To reveal the nodes having clinical relevance, we next linked the activity of each node with sample phenotypic information (Figure 1). The gene weights in each prioritized clinically relevant node were further investigated. Only the genes with either high-positive or high-negative weights were retained. The overlaps of high-weight genes among the prioritized nodes were chosen to develop the molecular signature (Figure 1). In this study, the hidden layer of DA was designed to contain 200 nodes, with epoch size of 1000, batch size of 200, corrupted level of 0.1, and learning rate of 0.01. We chose 200-node model in our study, since DA performance starts to be stable from 200 to 300 nodes, and increasing node size does not improve DA reconstruction when DA models are applied to genome-wide gene expression data [53]. To confirm the robust performance of DA model, we also ran a DA model with 400 hidden nodes and tested its predictive power. Because microarray data were used to train the DA model, genome-wide gene expression profiles were represented at probeset level. For each hidden node, high-weight genes/probesets were defined as those within either left or right 1% tail of the distribution of the weight vector.

### The training and validation data

To train the DA model, we collected 13 lung cancer related genome-wide gene expression datasets from the GEO [36] database (Table S1), which consisted of 1916 human lung tissue samples. All these datasets were based on Affymetrix Human Genome U133 Plus 2.0 Array. The GCRMA algorithm in Bioconductor was applied to normalize the expression level of each probeset for the microarray data. The function “mas5calls” in the Bioconductor “affy” package was used to estimate the present/absent status for each probeset. Only the probesets present in at least two third of the samples were retained. In total, 22,829 probesets were included in our training set. We further ranked the resulting expression values within each sample in ascending order. Finally, we linearly transformed the expression range of each probeset to be between 0 and 1 as suggested by Tan et al. [33].

Four validation datasets were included in this study, which were also obtained from the GEO database [36] and based on Agilent Homo sapiens 21.6K custom array, Affymetrix Human Genome U133 Plus 2.0 Array, CHUGAI 41K Array, and Rosetta/Merck Human RSTA Custom Affymetrix 2.0 microarray, respectively. The summarized gene expression data were obtained from the GEO Series Matrix files. For a gene with multiple probes/probesets, the geometric mean of all the probes/probesets mapping to the gene was used to measure the gene expression level.

### The *ADC*-index

We followed a scoring formula used in several previous studies [17], [56], [60], [63] to assign each human sample an *ADC*-score, which is a linear combination of weighted gene expression:

$$I_{\mathrm{ADC}}=\sum_{i=1}^{n}w_{i}\left(e_{i}-\mu_{i}\right)/\tau_{i}$$

Where *I_ADC_* is the *ADC*-index; *n* is the number of genes; *w_i_* is the weight of gene *i* (either 1 or −1 in this study); *e_i_* denotes the expression level of gene *i*; and *μ_i_* and *τ_i_* are the mean and standard deviation of the gene expression values for gene *i* across all samples, respectively.

### Statistical analyses

All the statistical analyses in this study were performed by the R platform. PCA was conducted by the “dudi.pca” function in the “ade4” library. *Cox* regression and log-rank test were performed by the “coxph” and “survdiff” functions in the “survival” library, respectively.

### PCA-based method to prioritize prognostic genes

PCA was conducted on our training data. We focused on the first 200 principal components, of which the first 30 components explained ~ 70% variation. In order to account for batch effect, we computed the difference among the components using one-way ANOVA. We only kept the components with *P* > 0.01 and finally got 148 components. Univariate *Cox* proportional hazards regression was used to examine the relationship between each component and ADC survival. The top five components with the strongest correlation with either overall or recurrence-free survival were retained. We next extracted the top 10% probesets that provided the strongest impact to these components, which can be uniquely mapped to 261 human genes.

## CRediT author statement

**Jun Wang:** Conceptualization, Methodology, Investigation, Writing - original draft. **Xueying Xie:** Methodology, Software, Formal analysis, Investigation, Writing - original draft. **Junchao Shi:** Software, Formal analysis, Investigation. **Wenjun He:** Investigation. **Qi Chen:** Investigation. **Liang Chen:** Investigation, Supervision, Writing - review & editing. **Wanjun Gu:** Conceptualization, Methodology, Investigation, Writing - review & editing, Funding acquisition. **Tong Zhou:** Conceptualization, Software, Formal analysis, Investigation, Data curation, Writing - review & editing, Project administration. All authors read and approved the final manuscript.

## Competing interests

The authors have declared no competing interests.

## Supplementary material

Supplementary figure S1 **Heatmap of node activity**. The top nodes distinguishing ADC patients from CTR, SCC, and LCC subjects were included. Red represents higher node activity while blue stands for lower node activity.

Supplementary figure S2 **The top nodes that are significantly associated with clinical outcome in ADC patients**. The recurrence-free survival data were analyzed here. The node activity of Node 52 and Node 193 (negative nodes) are decreased in ADC patients with poor recurrence-free survival. On the contrary, the activity of Node 187 and Node 195 (positive nodes) are increased in ADC patients with worse outcome.

Supplementary figure S3 **Correlation in probeset weight between the prioritized positive and negative nodes**. The orange dots represent the overlapping upregulated probesets, while the blue dots denote the overlapping downregulated probesets.

Supplementary data 4 ***Kaplan–Meier* curves for SCC (A) and LCC (B) patients in the validation cohorts**. Patients were stratified into two categories according to ADC-index. Orange curves are for the patients with high ADC-index while blue curves are for the patients with low ADC-index. High ADC-index patients are defined as those having an ADC-index greater than the median ADC-index. *P* values were measured by log-rank tests.

Supplementary data 5 **Robust performance of DA models**. Resampling test was conducted upon the pooled published prognostic genes (425 genes), 200-node DA genes (272 genes), 400-node DA genes (371 genes), and PCA genes (261 genes), respectively. The orange area shows the distribution of the mean of |*Z*| for the 1000 resampled gene signatures (with the identical size as the 35-gene signature) randomly picked up from the pool of the published prognostic genes. The blue and cyan areas show the distributions of the mean of |*Z*| for the 1000 resampled gene signatures randomly selected from the pool of the 200-node and 400-node DA genes, respectively. The purple area shows the distribution of the mean of |*Z*| for the 1000 resampled gene signatures randomly selected from the pool of the PCA genes. The black triangle stands for the mean of |*Z*| of the 35-gene signature.

Supplementary Table S1 **The Gene Expression Omnibus (GEO) series included in the training set**.

Supplementary Table S2 **The 35-gene signature**.
