# Supplementary figures and images for: Denoising Autoencoder, A Deep Learning Algorithm, Aids the Identification of A Novel Molecular Signature of Lung Adenocarcinoma
Source: Genomics Proteomics Bioinformatics. 2020 Dec 18;18(4):468–80. doi: 10.1016/j.gpb.2019.02.003 (PMC8242334; doi:10.1016/j.gpb.2019.02.003)

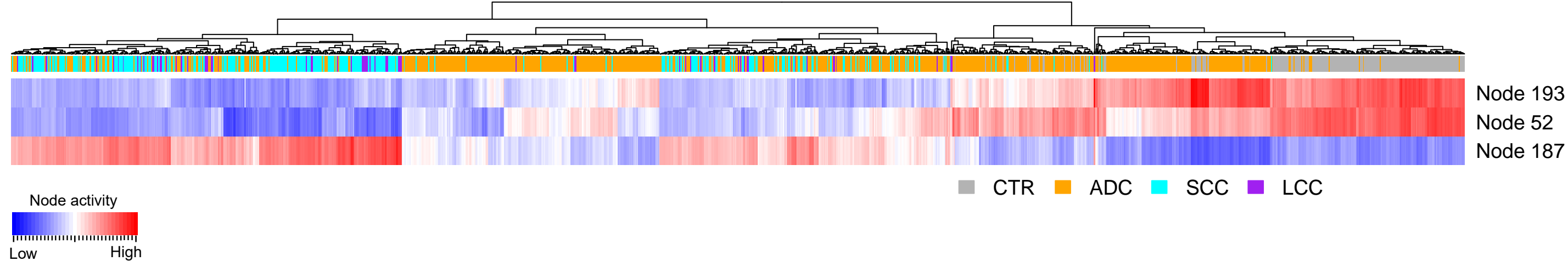

Supplement: Supplementary figure S1 — Heatmap of node activity. The top nodes distinguishing ADC patients from CTR, SCC, and LCC subjects were included. Red represents higher node activity while blue stands for lower node activity. [file mmc1.pdf]

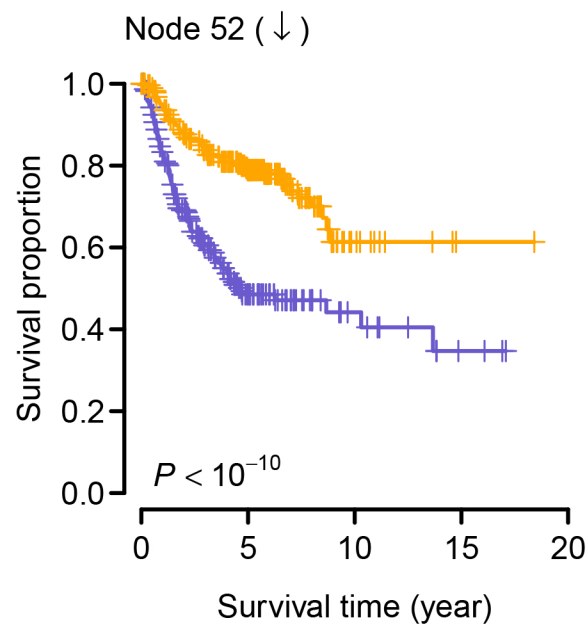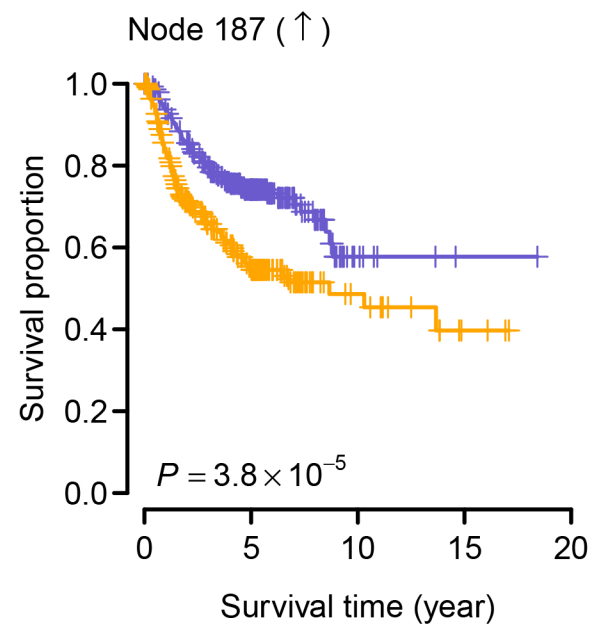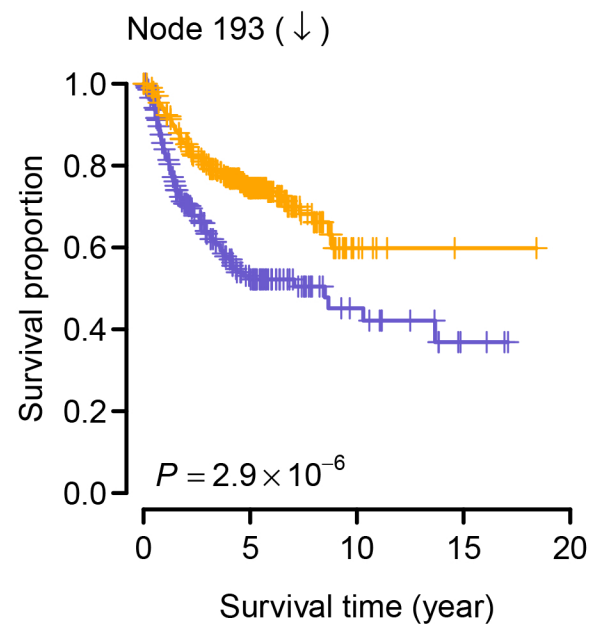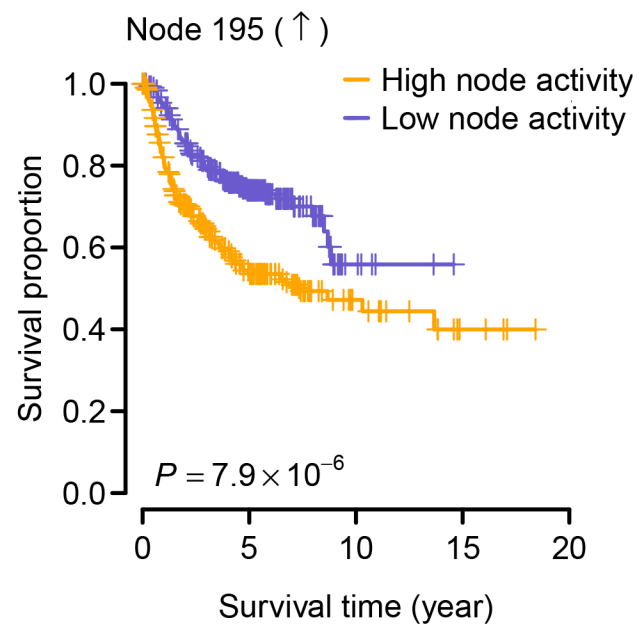

Supplement: Supplementary figure S2 — The top nodes that are significantly associated with clinical outcome in ADC patients. The recurrence-free survival data were analyzed here. The node activity of Node 52 and Node 193 (negative nodes) are decreased in ADC patients with poor recurrence-free survival. On the contrary, the activity of Node 187 and Node 195 (positive nodes) are increased in ADC patients with worse outcome. [file mmc2.pdf]

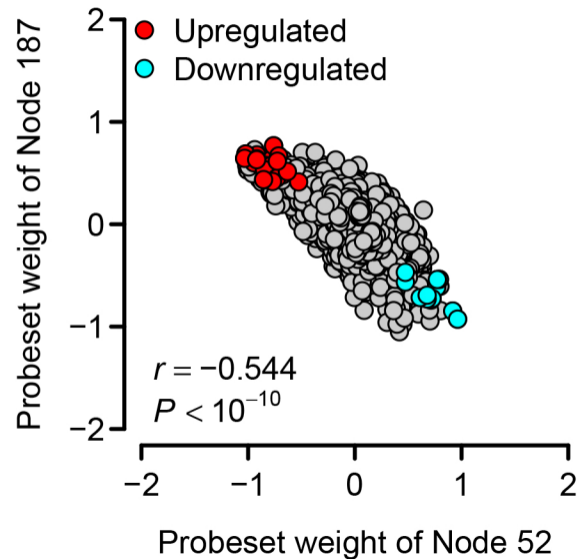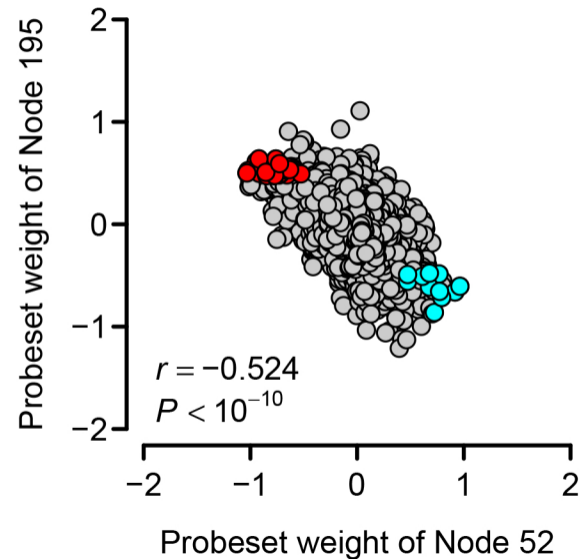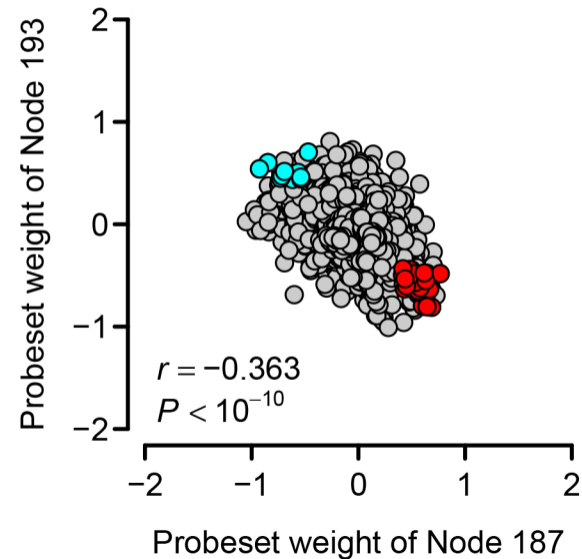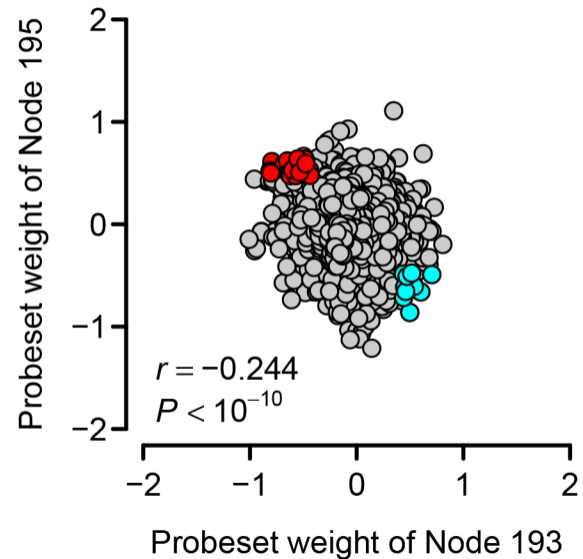

Supplement: Supplementary figure S3 — Correlation in probeset weight between the prioritized positive and negative nodes. The orange dots represent the overlapping upregulated probesets, while the blue dots denote the overlapping downregulated probesets. [file mmc3.pdf]

## A SCC

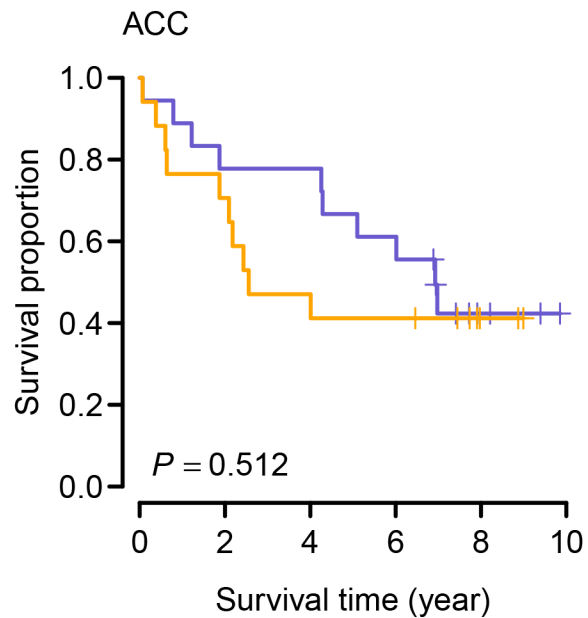

## B LCC

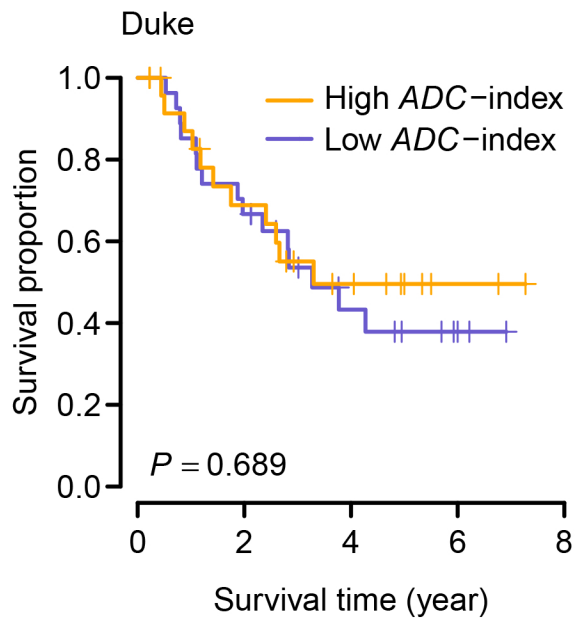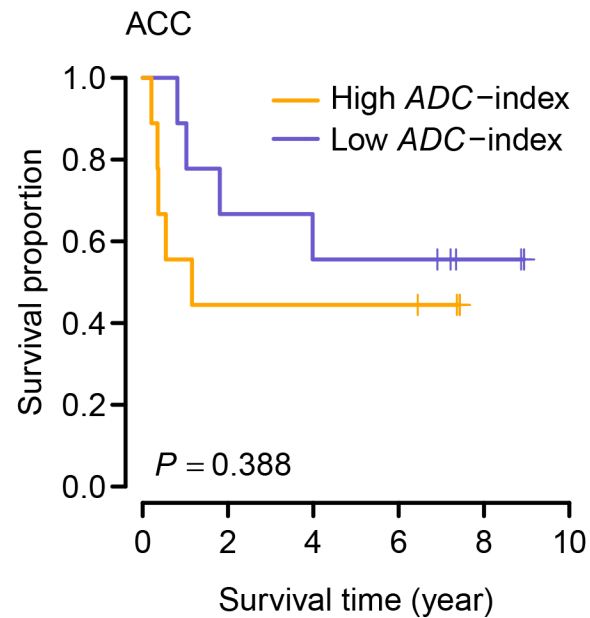

Supplement: Supplementary data 4 — Kaplan–Meier curves for SCC (A) and LCC (B) patients in the validation cohorts. Patients were stratified into two categories according to ADC-index. Orange curves are for the patients with high ADC-index while blue curves are for the patients with low ADC-index. High ADC-index patients are defined as those having an ADC-index greater than the median ADC-index. P values were measured by log-rank tests. [file mmc4.pdf]

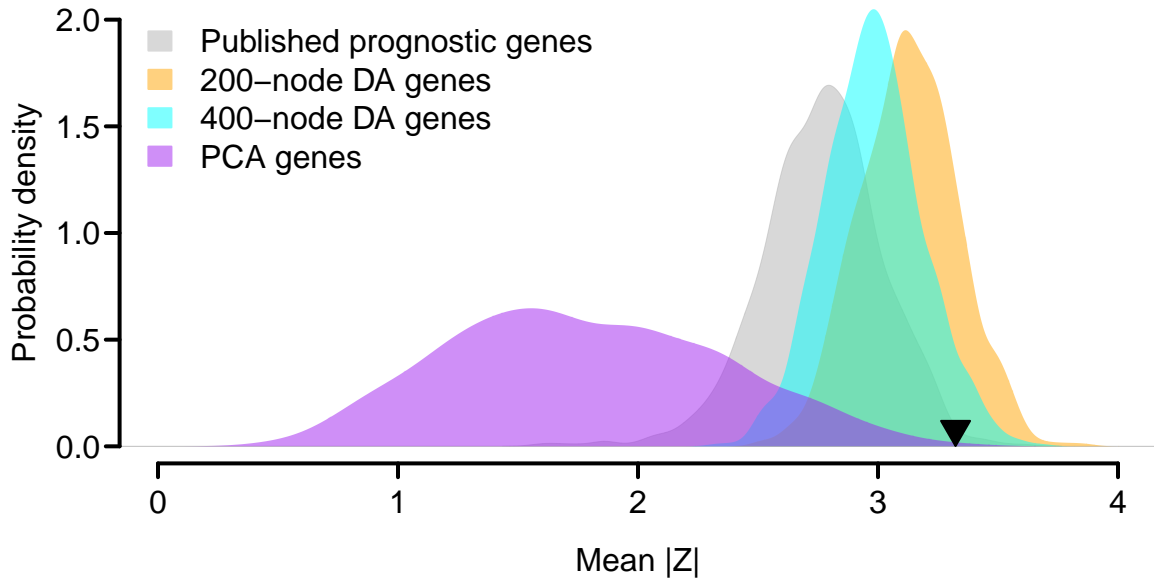

Supplement: Supplementary data 5 — Robust performance of DA models. Resampling test was conducted upon the pooled published prognostic genes (425 genes), 200-node DA genes (272 genes), 400-node DA genes (371 genes), and PCA genes (261 genes), respectively. The orange area shows the distribution of the mean of |Z| for the 1000 resampled gene signatures (with the identical size as the 35-gene signature) randomly picked up from the pool of the published prognostic genes. The blue and cyan areas show the distributions of the mean of |Z| for the 1000 resampled gene signatures randomly selected from the pool of the 200-node and 400-node DA genes, respectively. The purple area shows the distribution of the mean of |Z| for the 1000 resampled gene signatures randomly selected from the pool of the PCA genes. The black triangle stands for the mean of |Z| of the 35-gene signature. [file mmc5.pdf]
