# Supplementary material for: Denoising Autoencoder, A Deep Learning Algorithm, Aids the Identification of A Novel Molecular Signature of Lung Adenocarcinoma
Source: Genomics Proteomics Bioinformatics. 2020 Dec 18;18(4):468–80. doi: 10.1016/j.gpb.2019.02.003 (PMC8242334; doi:10.1016/j.gpb.2019.02.003)
Supplement: Supplementary Table S1 — The Gene Expression Omnibus (GEO) series included in the training set. [file mmc6.docx]

**Table S1 The Gene Expression Omnibus (GEO)** **series included in the training set**

| **GEO accession** | **Country** | **N** |
| --- | --- | --- |
| GSE10245 | Germany | 58 |
| GSE18842 | Spain | 91 |
| GSE19188 | Netherlands | 156 |
| GSE19804 | Taiwan | 120 |
| GSE28571 | Sweden | 100 |
| GSE29013 | USA | 55 |
| GSE30219 | France | 307 |
| GSE31210 | Japan | 246 |
| GSE33532 | Germany | 100 |
| GSE37745 | Sweden | 196 |
| GSE43580 | Switzerland | 150 |
| GSE50081 | Canada | 181 |
| GSE77803 | Denmark | 156 |
