# Supplementary material for: Denoising Autoencoder, A Deep Learning Algorithm, Aids the Identification of A Novel Molecular Signature of Lung Adenocarcinoma
Source: Genomics Proteomics Bioinformatics. 2020 Dec 18;18(4):468–80. doi: 10.1016/j.gpb.2019.02.003 (PMC8242334; doi:10.1016/j.gpb.2019.02.003)
Supplement: Supplementary Table S2 — The 35-gene signature. [file mmc7.docx]

**Table S2 The 35-gene signature**

| **Gene symbol** | **Gene title** | **Weight** |
| --- | --- | --- |
| *BIRC5* | baculoviral IAP repeat containing 5 | 1 |
| *BLM* | Bloom syndrome, RecQ helicase-like | 1 |
| *CCNB2* | cyclin B2 | 1 |
| *CDC6* | cell division cycle 6 homolog (*S. cerevisiae*) | 1 |
| *CDCA2* | cell division cycle associated 2 | 1 |
| *CDCA3* | cell division cycle associated 3 | 1 |
| *DEPDC1* | DEP domain containing 1 | 1 |
| *E2F7* | E2F transcription factor 7 | 1 |
| *FAM54A* | family with sequence similarity 54, member A | 1 |
| *FANCI* | Fanconi anemia, complementation group I | 1 |
| *FOXM1* | forkhead box M1 | 1 |
| *GINS1* | GINS complex subunit 1 (Psf1 homolog) | 1 |
| *GINS2* | GINS complex subunit 2 (Psf2 homolog) | 1 |
| *GTSE1* | G-2 and S-phase expressed 1 | 1 |
| *KIAA1524* | KIAA1524 | 1 |
| *MAD2L1* | MAD2 mitotic arrest deficient-like 1 (yeast) | 1 |
| *OIP5* | Opa interacting protein 5 | 1 |
| *PARPBP* | PARP1 binding protein | 1 |
| *PBK* | PDZ binding kinase | 1 |
| *PRC1* | protein regulator of cytokinesis 1 | 1 |
| *PRR11* | proline rich 11 | 1 |
| *PTHLH* | parathyroid hormone-like hormone | 1 |
| *RRM2* | ribonucleotide reductase M2 | 1 |
| *SLC2A1* | solute carrier family 2 (facilitated glucose transporter), member 1 | 1 |
| *SPC25* | SPC25, NDC80 kinetochore complex component, homolog (*S.* *cerevisiae*) | 1 |
| *TTK* | TTK protein kinase | 1 |
| *ADH1B* | alcohol dehydrogenase 1B (class I), beta polypeptide | –1 |
| *CYP4B1* | cytochrome P450, family 4, subfamily B, polypeptide 1 | –1 |
| *FOLR1* | folate receptor 1 (adult) | –1 |
| *MAMDC2* | MAM domain containing 2 | –1 |
| *NR3C2* | nuclear receptor subfamily 3, group C, member 2 | –1 |
| *PLA2G1B* | phospholipase A2, group IB (pancreas) | –1 |
| *SCN7A* | sodium channel, voltage-gated, type VII, alpha subunit | –1 |
| *SFTPC* | surfactant protein C | –1 |
| *VEPH1* | ventricular zone expressed PH domain homolog 1 (zebrafish) | –1 |
